# Engineering of pre-vascularized urethral patch with muscle flaps and hypoxia-activated hUCMSCs improves its therapeutic outcome

**DOI:** 10.1111/jcmm.12157

**Published:** 2014-01-25

**Authors:** Dongchong Sun, Yong Yang, Zhitao Wei, Yong Xu, Xu Zhang, Baofa Hong

**Affiliations:** Department of Urology, People's Liberation Army General HospitalBeijing, China

**Keywords:** hUCMSC, tissue engineering, urethral reconstruction, pedicled muscle flaps

## Abstract

Tissue engineering has brought new hopes for urethral reconstruction. However, the absence of pre-vascularization and the subsequent degradation of materials often lead to the failure of *in vivo* application. In this study, with the assistance of hypoxia-activated human umbilical cord mesenchymal stem cells (hUCMSCs), pedicled muscle flaps were used as materials and pre-incubated in ventral penile subcutaneous cavity of rabbit for 3 weeks to prepare a pre-vascularized urethral construct. We found that small vessels and muscle fibres were scattered in the construct after 3 weeks' pre-incubation. The construct presented a fibrous reticular structure, which was similar to that of the corpus spongiosum under microscope examination. The produced constructs were then used as a patch graft for reconstruction of the defective rabbit urethra (experimental group), natural muscular patch was used as control (control group). Twelve weeks after the reconstructive surgery, urethrography and urethroscope inspections showed wide calibres of the reconstructed urethra in the experimental group. Histopathological studies revealed that fibrous connective tissues and abundant muscle fibres constituted the main body of the patch-grafted urethra. In contrast, in the control group, only adipose tissue was found in the stenosis-reconstructed urethra, replacing the originally grafted muscular tissue. To our knowledge, this is the first report that successfully constructed a pre-vascularized urethral construct by using hypoxia-activated hUCMSC and pedicled muscle flaps. More importantly, the pre-vascularized construct showed a good performance in urethral reconstruction when applied *in vivo*. The study provided a novel strategy for tissue engineering of pre-vascularized urethral construct for the defective urethra, representing a further advancement in urethral reconstruction.

## Introduction

Urethral reconstruction is a long-standing issue in reconstructive urological surgery, and the vascularization of the grafts used is still a critical factor influencing the success of urethral reconstruction [1–2]. In recent years, development of tissue engineering materials has been brought to the forefront of reconstructive urology as a way to improve the reconstructive effects [4–5]. Many natural and synthetic materials have been developed for urethral construction, such as collagen [7–8]. However, when it comes to the *in vivo* application for urethral reconstruction, the degradation of these reconstructive materials and the blood supply are still problems. In fact, we still have few ‘living’ bioengineered materials for urethral reconstruction.

Pedicled skin or muscle flaps provided durable and stable materials for tissue reconstruction. They possess complex architecture of native tissues which are still impossible to construct by artificial effort. Therefore, these pedicled flaps have been extensively investigated for the reconstruction of various soft tissue defects [10–11]. As native tissues, these flaps are abundant of vascular progenitor cells and intrinsic vasculatures which would migrate and form new vasculatures of reconstructed tissues when activated by *in vivo* angiogenic conditions, such as VEGF. However, endogenous angiogenic cytokines are usually insufficient to support the vasculature formation in tissue construct, severely influencing the vascularization and quality of reconstructed tissues, as well as their therapeutic outcomes in tissue repair [13, 14]. Exogenously supplementing cytokines would be helpful in promoting vascularization in the construct, but the lifecycle of the cytokines is too short to sustain long-term activity.

Mesenchymal stem cells (MSCs) are widely used as seeding cells in tissue engineering. It has been definitely demonstrated that MSCs possessed a robust paracrine capacity for angiogenic cytokines and other tropic factors [15, 16]. Many studies have used MSCs for improving tissue neovascularization or growth, and achieved encouraging results [17–18]. In addition, indifferent groups have confirmed that stress conditions, such as hypoxia [15], could dramatically enhance the secretion of angiogenic cytokines from MSCs, which is of great significance in regenerative medicine.

**Figure 1:**
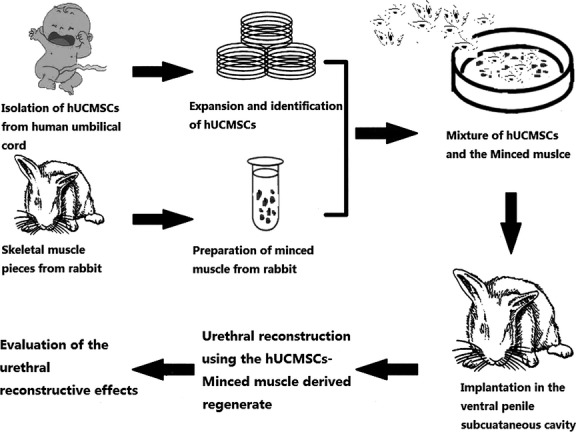
Overview of the experimental procedures.

We suggest that mixing hypoxia-pretreated MSCs with pedicled muscle flaps to prepare a urethral construct would significantly improve the quality of constructed tissues. Hypoxia-pretreated MSCs will provide abundant angiogenic cytokines required for vascularization. More importantly, the auxo-angiogenic effects will be sustained as long as the MSCs survived. In this study, we aimed to construct a pre-vascularized living patch graft for urethral reconstruction *in vivo* using a mixture of autologous minced skeletal muscle and hypoxia-pretreated human umbilical cord MSCs (hUCMSCs). These tissues and cells were first pre-incubated in the ventral penile subcutaneous cavity of the rabbit to mature into a pre-vascularized living construct and then transplanted as an autologous patch graft for the reconstruction of the rabbit urethra. The feasibility and effectiveness of this strategy for urethral reconstruction were systematically investigated. The experimental procedures are illustrated in Figure 1.

## Materials and methods

### General information

Altogether, a total of 28 healthy male New Zealand rabbits were used in this study, with 21 and 7 in the experimental and control groups, respectively. The animals were treated under the Guide for the Care and Use of Laboratory Animals [20]. All the surgical procedures were conducted under sterile conditions with intramuscular anaesthesia consisting of Sumianxin II (0.3 ml/kg; Military Veterinary Institute, China).

### Isolation, cultivation and characterization of hUCMSCs

With the consent of the parents, fresh umbilical cords were collected according to the regulations of the Chinese Academy of Medical Sciences and the Research Ethics Committee of the General Hospital of People's Liberation Army. Isolation, cultivation and characterization of hUCMSCs were conducted as previously described in the literature [21]. Flow cytometry was performed to determine the immunophenotype of the cells. Multipotency of hUCMSCs was confirmed by osteogenic and adipogenic differentiation.

### Elisa analysis

10^6^ hUCMSCs were seeded in culture plates and cultured for 24 hrs in normoxic (21% O_2_) or hypoxic (1% O_2_) conditions. The culture media were then collected and analysed for growth factors, VEGF, HGF and IGF-1 by ELISA array. Data are expressed as mean ± SEM pictograms of the secreted factor/10^6^ hUCMSCs.

### Preparation of skeletal muscle fragments

After anaesthesia, a piece of skeletal muscle (about 100 mg) was harvested from the left hind limb of each rabbit and temporarily kept in Dulbecco's modified Eagle's medium at room temperature. The wound was closed with interrupted sutures. After being washed and weighed, the muscle piece was minced with scissors into fragments of no more than 1 mm in diameter.

### Preparation of muscle and hUCMSCs-derived urethral constructs

Implantation of the minced muscle and hUCMSCs inside the ventral penile subcutaneous cavity was conducted as follows: after re-anaesthesia, a 0.5-cm incision was made in the ventral penile skin of the rabbit. By blunt dissection, a subcutaneous cavity was created. Muscle fragments and hypoxia-pretreated (24 hrs) hUCMSCs were mixed (2 × 10^6^ hUSMSCs/100 mg fragments). The mixture was then injected inside the ventral penile subcutaneous cavity and the injected volume can be adjusted to acquire the constructs of different sizes. The incision was closed with interrupted silk sutures. The rabbits were left to recover and then return to normal breeding facilities.

### RT-PCR

Total RNA of cells and tissues was extracted with RNAprep pure Cell/Bacteria Kit (TIANGEN, Beijing, China) according to manufacturer's instruction. Reverse transcription was performed with standard procedures to synthesize first-strand cDNA. Human sequence-specific primers for VEGF, HGF and IGF-1 were used in the following PCR reaction as the previous report [22].

### Western blotting

Western blotting analysis was performed according to the previous reports [22, 23]. Briefly, tissues were lysed in Laemmli Sample Buffer (Bio-Rad, Hercules, CA, USA) and were further homogenized with a rotor stator homogenizer. Proteins were collected and the protein content was determined with BCA Protein Assay Kit (Thermo Scientific, Waltham, MA, USA). After electrophoresis, proteins were transferred to a PVDF Western blotting membrane (Roche, Basilea, Switzerland). Anti-human VEGF, HGF and IGF-1 antibodies were used for detection, GAPDH was used as the internal standard.

### Urethral reconstruction with hUCMSCs and muscle fragment-derived constructs

In the experimental group, after 3 weeks of pre-incubation inside the ventral penile subcutaneous cavity, a second surgery was performed. Along the interface between the hUCMSC and minced muscle-derived construct and the ventral urethra, a dissection was made to release the construct, whereas the interface between the ventral penile skin and the construct was kept untouched. With fine scissors, a small piece of the construct was removed and kept in a −80°C refrigerator for later histopathological evaluation. The construct was carefully tailored into a 0.5 × 0.5 cm round patch. After a round ventral urethral defect of 0.5 × 0.5 cm was made in posterior urethra, the margin of the urethral defect was sutured to the construct with six interrupted polygalactin (Vicryl) 7-0 sutures. The incision was closed with an interrupted 4-0 Vicryl stitch. Neither stent nor dressing was used. After the surgery, the animals were left to recover freely.

In the control group, because no construct could be formed when the minced muscle was implanted inside a subcutaneous cavity without hUCMSCs (unpublished data from our preliminary experiments), we selected to take only a piece of skeletal muscle patch (0.5 × 0.5 × 0.3 cm) from the rabbit's left hind limb as an autologous patch graft for the urethral reconstruction.

### Post-operative observations and histopathological evaluations

Biopsies from the hUCMSC-and minced muscle-derived construct were prepared into serial 5-μm frozen sections for haematoxylin–eosin staining and immunohistochemical studies. Mouse anti-desmin (DE-U-10; Abcam, Cambridge, UK), mouse anti-human specific nuclei antigen (MAB1281; Millipore, Billerica, MA, USA), and mouse anti-CD31/PECAM1 monoclonal antibodies (NB600-562; Novus Biologicals, Littleton, CO, USA) were used as the primary antibodies, and goat antimouse IgG, FITC or RBITC-conjugated goat antimouse IgG monoclonal antibodies were used as the secondary antibodies, respectively. Control sections omitted the primary antibody and were treated with PBS alone. Observations on these samples were conducted using a light microscope or laser scanning confocal microscope.

After the reconstructive surgery, the rabbits in the experimental group were subdivided equally into three groups of seven each and killed 2, 4 and 12 weeks after the urethral reconstructive surgery with an overdose of anaesthesia. At each scheduled time-point, the entire penis of the rabbit was examined, removed, fixed in 10% formaldehyde and processed for haematoxylin–eosin and Masson's trichrome staining.

Retrograde urethrography and urethroscope observations were conducted just before the final autopsy. For the control group, urethral inspections and autopsies were taken only 12 weeks after the reconstructive surgery.

## Results

### Characterization of hUCMSCs

**Figure 2:**
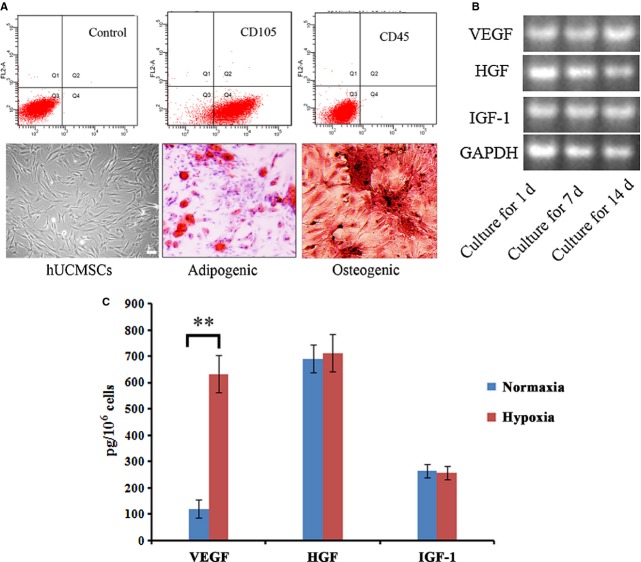
Isolation and characterization of human umbilical cord mesenchymal stem cells (hUCMSCs). (A) Immunophenotype analysis by flow cytometry and multi-differentiation of hUCMSCs; (B) RT-PCR analysis of cytokine gene expression; (C) ELISA analysis of cytokine secretion from hUCMSCs in normaxia and hypoxia condidions. **P < 0.01. Bar = 100 μm. hUCMSCs indicated human umbilical cord mesenchymal stem cells.

The freshly isolated hUCMSCs appeared round in shape. When seeded in culture dishes, the cells were adherent, elongated and showed spindle-shaped. Flow cytometry analysis demonstrated that most of hUCMSCs are positive for CD105, CD44 and negative for CD31, CD45. When cultured in osteogenic and adipogenic conditions, hUCMSCs successfully differentiated into osteocytes and adipocytes, which were positive for alizarin red and Oil Red O staining, respectively (Fig. 2A).

We further analysed the paracrine capacity by RT-PCR, several factors that play important roles in angiogenesis were selected, including VEGF, HGF and IGF-1. hUCMSCs maintained a high expression of VEGF, HGF, IGF-1 in mRNA level during *in vitro* long-time cultivation(up to 14 days, Fig. 2B). ELISA analysis demonstrated hypoxia pre-treatment could significantly reinforce the paracrine capacity of hUCMSCs for VEGF (more than five times as normaxia condition, Fig. 2C).

### Sustained angiogenic cytokine release of hUCMSCs in urethral construct *in vivo*

**Figure 3:**
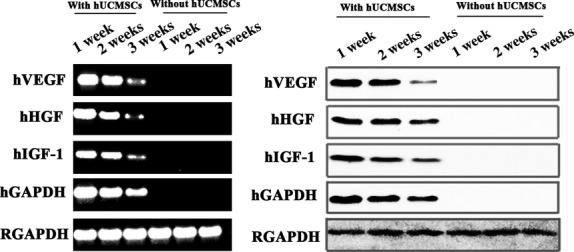
RT-PCR and Western blotting analysis of sustained cytokine release from human umbilical cord mesenchymal stem cells *in vivo*. Human sequence-specific primers for VEGF, HGF and IGF-1 were used for RT-PCR analysis of urethral construct; Anti-human antibodies against VEGF, HGF and IGF-1 were used in Western blotting.

During *in vivo* cultivation, mixed constructs (prepared by muscle fragments and hUCMSCs) and control constructs (pure muscle construct) were collected at 1, 2 and 3 weeks for RT-PCR and Western blotting analysis. We took advantage of human-specific sequences and antibodies as the previous report [22] to detect cytokines secreted by hUCMSCs mixed in construct. As shown in Figure 3, RT-PCR demonstrated that the human angiogenic cytokine genes were persistently expressed during *in vivo* cultivation of mixed construct, indicating that hUCMSCs survived during the whole process and continually secreted angiogenic cytokines. Furthermore, the secretion of human angiogenic cytokines in mixed constructs was also demonstrated by Western blotting using human-specific antibodies. In control constructs, both RT-PCR and Western blotting did not detect the expression of these cytokines.

### Characteristics of construct derived from hUCMSC and muscle fragments

**Figure 4:**
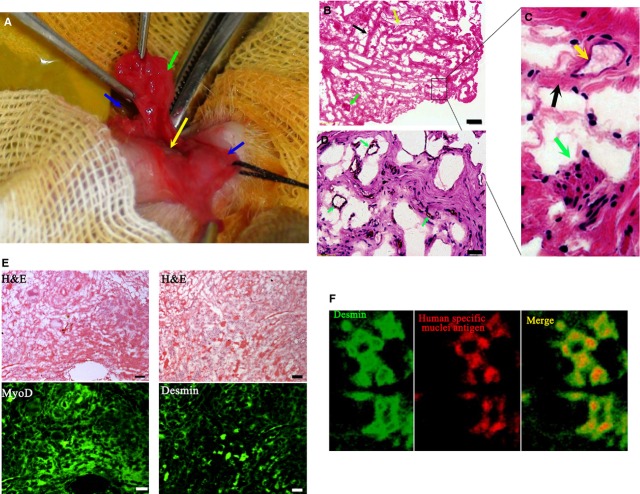
Characterization of urethral construct collected at 3 weeks after pre-incubation inside the subcutaneous ventral penile cavity. (A) The construct. Green arrow indicates the body of the construct, the blue arrow indicates the foreskin (right side), the yellow arrow indicates the urethra and the black arrow indicates the foreskin (left side). (B) Reticular structures distributed with fibrous bundles (black arrow), small vessels (yellow arrow) and muscle fibres (green arrow) found in the construct under the microscope (bar = 100 μm). (C) Magnification of the structures from B (D) Vessel structure in the construct under the microscope (bar = 100 μm). (E) Muscle cell nature of the construct. Paraffin-embodied specimen of construct was prepared. Continuous sections were then prepared and stained by haematoxylin–eosin staining or against muscle markers, MyoD and Desmin (fluorescent images, bar = 100 μm). (F) Co-expression of desmin and human-specific nuclei antigen in the human umbilical cord mesenchymal stem cells-and minced muscle-derived regenerate under a laser scanning confocal microscope. MyoD indicates myogenic differentiation antigen.

After 3 week pre-incubation *in vivo*, constructs were collected. In rabbits receiving implantation of mixed constructs, a solid oblatoid construct was found in the ventral penile subcutaneous cavity of the rabbits. There was a relatively tight adhesion of the construct to the foreskin side. After release, the construct presented a rubicund patch-like shape (Fig. 4A). Haematoxylin–eosin staining showed a reticular structure in the construct. Fibrous connective tissues with some scattered muscle cells were observed in haematoxylin–eosin-stained sections too (Fig. 4B and C). Small blood vessels or sinusoids were scattered throughout the construct (Fig. 4C and D). These results suggested that hUCMSCs played a potent role in pre-vascularization of construct. Furthermore, immune-fluorescent staining demonstrated that cells with muscle morphology in haematoxylin–eosin sections were positive to muscle markers, including MyoD and Desmin, confirming muscle cell nature of the construct (Fig. 4E). Immune-fluorescent staining also revealed the co-expression of desmin and human-specific nuclei antigen at the periphery of the mixed construct, indicating the myogenic differentiation of hUCMSCs (Fig. 4F).

In comparison, in rabbits receiving implantation of muscle fragments, no construct of certain shape was formed. Therefore, no further detection was performed.

### Effects of pre-vascularized construct on urethral reconstruction

**Figure 5:**
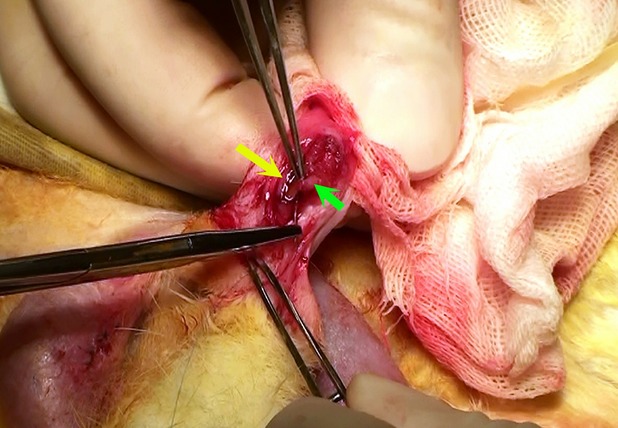
A view of the surgical procedures during urethral reconstruction with the human umbilical cord mesenchymal stem cells-and minced muscle-derived construct. The green arrow indicates the body of construct and the yellow arrow indicates the margin of the urethral defect.

The urethral reconstructive surgeries proceeded uneventfully in all the rabbits. There were no muscle harvest-associated complications. The pre-vascularized constructs showed excellent flexibility and toughness that was adequate when used as a patch graft in the reconstructive surgery (Fig. 5). Post-operative observations showed that all the animals survived their intended survival period with no evidence of serious haematuria, urinary infections, voiding difficulties or fistulae formations. Because of no construct of certain shape was formed by pure muscle fragments in construct preparation, we selected natural skeletal muscle patch for urethral reconstruction as control.

#### Ureteroscope and urethrography examinations

**Figure 6:**
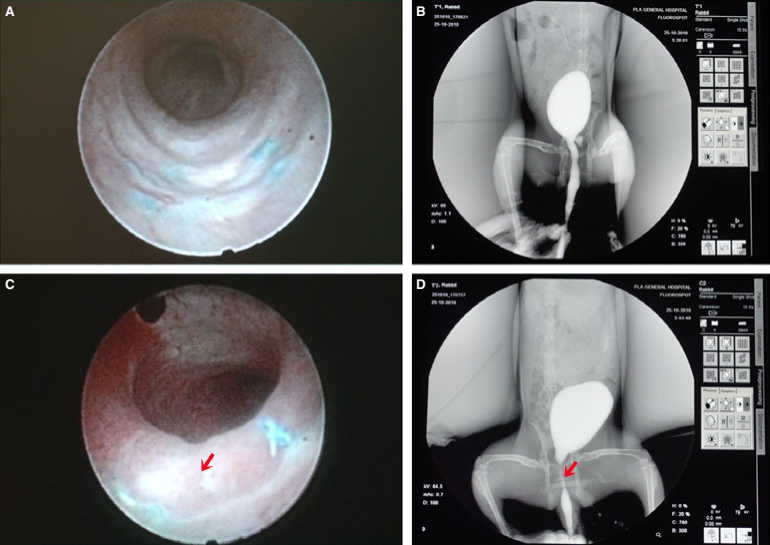
Ureteroscope and urography observations of the reconstructed urethra 12 weeks after the reconstructive surgery. (A) Ureteroscope observations in the experimental group; (B) urography observations in the experimental group; (C and D) urography observations in the control group. The red arrows indicate urethral stenosis.

At 12 weeks after the reconstructive surgery, urethroscope (Fig. 6A) and urethrography (Fig. 6B) examinations showed a wide urethral calibre in the experimental group rabbits with no signs of obvious stricture, fistula or pseudo-diverticulum. However, in the control group (Fig. 6C and D), typical urethral strictures were found in most animals (five of seven rabbits).

#### Histology

**Figure 7:**
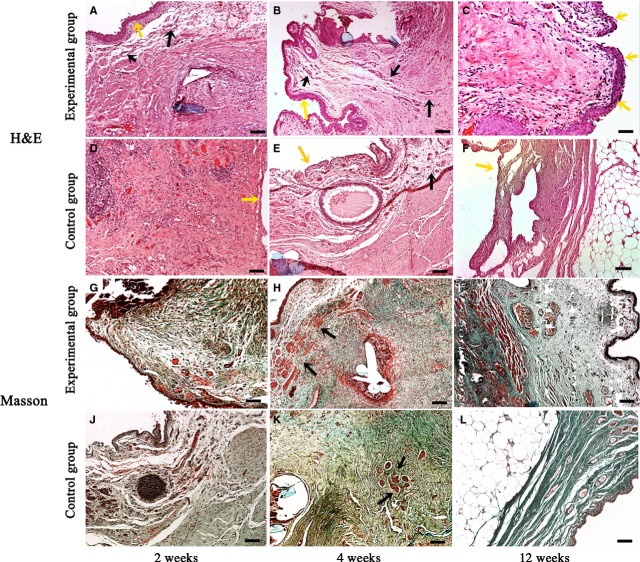
Histological inspections of the reconstructed urethra after reconstructive surgery. (A–C) Haematoxylin–eosin staining at 2, 4 and 12 weeks from the experimental group. As early as 2 weeks, the grafted urethra was found to be covered with urothelium (yellow arrows in A and B). Small vessels (black arrows in A and B) and muscle fibres were distributed dispersedly; good morphology of reconstructed tissues was observed at 12 weeks (C); (D–F) haematoxylin–eosin staining at 2, 4 and 12 weeks from the experimental group. No obvious urothelium was observed in the control graft (yellow arrows in D and E); vessel density was obviously lower than that in the experimental group (black arrows in E); grafted muscle was largely substituted by adipose tissues in the reconstructed urethra at 12 weeks (F); (G–L) Masson's trichrome staining at 2, 4 and 12 weeks. Abundant muscle tissues were observed in the experimental group (G–I, red staining), whereas muscle tissues were much less in the control group (J–L, red staining). Especially at 12 weeks, most tissues were substituted by collagen (green staining); bar = 100 μm.

At 2, 4 and 12 weeks after the urethral reconstructive surgery, haematoxylin–eosin staining showed that the grafted urethra in experimental group was covered with urothelium as early as 2 weeks, lots of vessel structures were formed and no significant necrosis was observed throughout the 12 weeks (Fig. 7A–C). In contrast, though similar morphology was observed between experimental group and control group at 2 weeks (Fig. 7A and D), obviously less vessel structures were formed with time in control group than that in experimental group (Fig. 7B and E). At 12 weeks, good morphology of reconstructed tissues was still observed in experimental group (Fig. 7C). However, the grafted muscle patch in control group had already been largely substituted by adipose tissues (Fig. 7F).

Masson's trichrome staining at 2, 4 and 12 weeks provided additional evidence that the urethral constructs we prepared have good performance *in vivo* for urethral reconstruction. As shown in Figure 7G–I, abundant muscle tissues were observed in experimental group (especially from 4 weeks, red staining in Masson's trichrome stained sections), whereas muscle tissues were much less in control group (Fig. 7J–L). Especially at 12 weeks, most tissues in control group were substituted by collagen (Fig. 7L, green staining).

To determine the role of hUCSMCs in urethral reconstruction, we further detected the expression of human-specific nuclei antigen at 2, 4 and 12 weeks, only a few positive cells were observed at 2 weeks, no such cells were detected at 4 or 12 weeks (data not shown). The results indicated that the good performance of the urethral construct in urethral reconstruction was independent of the long-term survival or transdifferentiation of hUCSMCs, that is, the paracrine effect may be a crucial mechanism in the process.

## Discussion

For stem cell-based tissue engineering for urethral reconstruction, a successful strategy depends on the correct selection and revascularization of the reconstructive materials. An autologous pedicled tissue is naturally one of the most ideal carrying materials for stem cells. In this study, we firstly prepared a pre-vascularized urethral construct by using hypoxia-activated hUCMSCs and pedicled muscle flaps. Furthermore, we demonstrated that the pre-vascularized construct showed a good performance in urethral reconstruction when applied *in vivo*.

Adult bone marrow stem cells are usually considered the major source of MSCs for cell therapy. However, aspiration of bone marrow stem cells involves invasive procedures. In comparison, hUCMSCs have fewer ethical issues, easy availability, an abundant source and demonstrate rapid proliferation and versatile differentiation potentials [24]. hUCMSCs also have lower expression of MHC class I (major histocompatibility complex calss I, MHC I) and negative expression of MHC class II (MHC II)[25]. A comparative study has indicated that hUCMSCs are an excellent alternative to bone marrow stem cells as a source of MSCs for cell therapies [26]. Therefore, hUCMSCs were selected in the study.

In our primary studies, we found that the minced muscle alone could not regenerate in a non-muscular environment. However, with the addition of hUCMSCs, a concrete neo-tissue could be regenerated from the minced muscle in the ventral penile subcutaneous cavity. This laid the foundation for us to use this stem cell-assisted, minced muscle-derived construct as an autologous urethral reconstructive material. We found no direct evidence in the literature concerning interactions of hUCMSCs with minced muscle. Adipose-derived stem cells have been reported to have supportive potentials for the survival of fat tissues when transplanted together [27, 28], and hUCMSCs also have the ability to synthesize and secret a set of tropic factors and cytokines that support the expansion and function of other cells (*i.e*., haematopoietic stem cells, embryonic stem cells, natural killer cells, islet-like cell clusters, neurons and glial cells) [29]. Therefore, we reasonably attributed the stromal supporting effects of hUCMSCs to the development of these minced muscle-derived constructs.

Conconi *et al*. confirmed the myogenic differentiation potential of hUCMSCs by direct injection of hUCMSCs into animal muscles [30]. In our study, desmin-positive muscle cells were also found in the hUCMSCs–-and minced muscle-derived constructs, and morphologically, the presence of central nuclei suggested their neonatal status. Together with the laser scanning confocal microscopic findings indicating the identical location of positive desmin and human-specific nuclei antigen, we concluded that some hUCMSCs had differentiated into muscle cells under these circumstances. However, this does not necessarily mean that all the muscle cells differentiated from hUCMSCs will naturally become the major constituent of the patch graft in the reconstructed urethra.

In fact, some researchers have reported that, in spite of the fact that hUCMSCs have low expression of MHC I and are negative for MHC II, hUCMSCs can still be activated to increase MHC I and to express MHC II following IFN-γ stimulation (especially following repeated application) or in inflammatory environments [25]. In this study, hUCMSCs were heterogeneous to the rabbit, and the minced muscle would likely go through a necrotic and inflammatory process. Together, these would inevitably lead to a compromised survival and differentiation of hUCMSCs. This presents a reasonable explanation to the negative findings of human-specific nucleic antigen in the grafted urethra when inspected 12 weeks after the reconstructive surgery.

In the study group, wide urethral calibres without obvious stenosis or fistulae were found in all animals. However, absence of the corpora spongiosum in the reconstructed urethra is a limitation of this study. The reconstruction of the corpora spongiosum tissue is undoubtedly of great significance to the functional and morphological restoration of the urethra. However, microscopically, we found reticular structures in the minced muscle-derived construct with scattered small vascular structures. This indicated that these constructs had not only been well revascularized but also formed porous structures, which might represent a primitive spongeous tissue from a tridimensional perspective. Still, the construction of the corpora spongiosum is a main object of our future work.

In conclusion, using the ventral penile subcutaneous cavity as a bioreactor and with the support of hypoxia-pretreated hUCMSCs, the minced muscle-derived construct could be effectively generated and used as a living patch graft for urethral reconstruction. This stem cell-based, muscle repair-oriented reconstructive strategy might lead to promising outcome for future urethral reconstruction.
